# *De novo* sequencing and analysis of the cranberry fruit transcriptome to identify putative genes involved in flavonoid biosynthesis, transport and regulation

**DOI:** 10.1186/s12864-015-1842-4

**Published:** 2015-09-02

**Authors:** Haiyue Sun, Yushan Liu, Yuzhuo Gai, Jinman Geng, Li Chen, Hongdi Liu, Limin Kang, Youwen Tian, Yadong Li

**Affiliations:** College of Horticulture, Jilin Agricultural University, Changchun, Jilin China

**Keywords:** Cranberry, Illumina sequencing, Fruit transcriptome, Flavonoid

## Abstract

**Background:**

Cranberries (*Vaccinium macrocarpon* Ait.), renowned for their excellent health benefits, are an important berry crop. Here, we performed transcriptome sequencing of one cranberry cultivar, from fruits at two different developmental stages, on the Illumina HiSeq 2000 platform. Our main goals were to identify putative genes for major metabolic pathways of bioactive compounds and compare the expression patterns between white fruit (W) and red fruit (R) in cranberry.

**Results:**

In this study, two cDNA libraries of W and R were constructed. Approximately 119 million raw sequencing reads were generated and assembled *de novo*, yielding 57,331 high quality unigenes with an average length of 739 bp. Using BLASTx, 38,460 unigenes were identified as putative homologs of annotated sequences in public protein databases, including NCBI NR, NT, Swiss-Prot, KEGG, COG and GO. Of these, 21,898 unigenes mapped to 128 KEGG pathways, with the metabolic pathways, secondary metabolites, glycerophospholipid metabolism, ether lipid metabolism, starch and sucrose metabolism, purine metabolism, and pyrimidine metabolism being well represented. Among them, many candidate genes were involved in flavonoid biosynthesis, transport and regulation. Furthermore, digital gene expression (DEG) analysis identified 3,257 unigenes that were differentially expressed between the two fruit developmental stages. In addition, 14,473 simple sequence repeats (SSRs) were detected.

**Conclusions:**

Our results present comprehensive gene expression information about the cranberry fruit transcriptome that could facilitate our understanding of the molecular mechanisms of fruit development in cranberries. Although it will be necessary to validate the functions carried out by these genes, these results could be used to improve the quality of breeding programs for the cranberry and related species.

**Electronic supplementary material:**

The online version of this article (doi:10.1186/s12864-015-1842-4) contains supplementary material, which is available to authorized users.

## Background

The American, or large cranberries (*Vaccinium macrocarpon* Ait), renowned for their health benefits, are an important berry crop [1]. Today, cranberries are commercially cultivated in the USA, Canada, Chile, Europe and China. In 2012, 504,030 tonnes were produced worldwide [2]. Most commercial cranberries produce round and red fruits, which are useful for fresh and frozen products, juice, wine, fruit beverages, jelly and jam. Cranberries are a popular fruit because of their attractive color, special flavor and nutritional values [3].

Their nutritional quality is mainly determined by the production and accumulation of bioactive compounds. The bioactive compounds of *V. macrocarpon* have been analyzed previously in fresh fruit and cranberry juice by high-performance liquid chromatography (HPLC), ultraviolet–visible (UV/Vis) and mass spectrometer (MS) detection [4–6]. There have been 10,038 phytochemicals detected in cranberry metabolomics profiles [6]. The major bioactive compounds in cranberries are flavonoids, including anthocyanins, proanthocyanidins (PAs), flavonols, flavan-3-ols (catechins), and a series of phenolic acid derivatives [7]. These phytochemicals have high antioxidant potential and beneficial health properties, including the prevention and treatment of cardiovascular diseases, various cancers, obesity, and infections involving the urinary tract, dental health, and *Helicobacter pylori*-induced stomach ulcers and cancers [8–17].

The cranberry fruit is an accessory fruit that develops from the development of the inferior ovary, which consists of the ovary wall and the floral tube [18]. Fruit ripening has an impact on the levels of various phytochemicals, such as vitamins, flavonoids and the phenolic acid derivatives. Hence, the study of the growth and ripening of cranberry fruits is an important field of research, as it influences the quality of the fruit, affecting pigment accumulation, flavor, nutritional value, and functionality. In particular, pigment accumulation during fruit ripening takes place from a bright green to white, then pink, and finally red, conferring the natural pigmentation to mature fruits [19, 20]. Given the distribution of these phytochemicals vary differently among different plants, even within different populations of the same species and different organs of plants, for this reason the molecular mechanisms of their biosynthesis, transport and regulation might be diverse and complex. Therefore, it is essential to use modern genetic tools for dissect out the complexities involve with phytochemicals in cranberry.

There has been some previous research into the cranberry, including simple sequence repeats (SSRs) mining and validation, genetic map construction, and quantitative trait loci (QTL) analysis [21–23]. Recently, a sequencing of one cultivar of the cranberry was published [24]. However, information about the development of the fruit of the cranberry is still scarce. The application of next-generation sequencing (NGS) technologies such as deep-sequencing dependent RNA sequencing (RNA-Seq), in particular *de novo* sequencing, provides a cost-effective means for sequencing the transcriptome of an organism [19–23]. Among the new-generation sequencing methods, 454 pyrosequencing techniques and Illumina sequencing are widely used to analyze transcriptomes [25–29]. Compared with 454 pyrosequencing, Illumina HiSeq 2000 costs less and has a much greater output. This makes HiSeq 2000 an enabling approach for high-throughput RNA-Seq. HiSeq 2000 sequencing technology has become a valuable tool for the study of fruit trees, such as the black raspberry [30], *Lycium chinense* [31], and the longan [32].

In this work, we present a *de novo* assembly of the fruit transcriptome of *V. macrocarpon* using high-throughput Illumina HiSeq 2000 sequencing. Furthermore, differential gene expression between white and red fruits was investigated to reveal differential regulation of key pathways. This study provides an important genetic resource for understanding molecular mechanisms on the cranberry fruit ripening, and the results may be helpful for further gene expression and functional genomic studies, and molecular breeding of the cranberry.

## Results and discussion

### cDNA sequence generation, *de novo* assembly and mapping to the cranberry genome

**Fig. 1:**
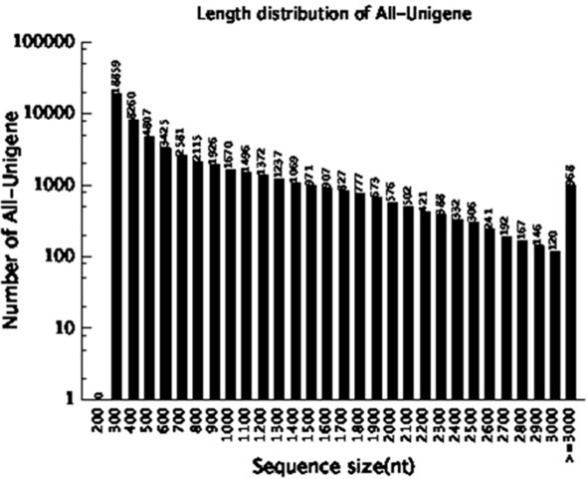
Length distribution of All-unigenes in cranberry fruit transcriptome

To obtain a complete profile of the cranberry transcriptome during fruit development, two cDNA libraries were built for two fruit developmental stages: white fruit (W) and red fruit (R). 59,986,374 and 59,690,570 raw reads were generated from the W and R libraries, respectively. A summary of these sequencing results are presented in Table 1. After removing low quality short sequences, 52,413,112 and 53,352,808 clean reads for W and R, respectively, remained and were used for assembly. The Q20 percentages (sequencing error rate <1 %) and GC percentages obtained from the W and R libraries were 97.86 % and 46.93 %, and 97.93 % and 47.24 %, respectively. These results suggest that the sequencing data have sufficient quantity and quality to ensure accurate sequence assembly and adequate transcriptome coverage. Cleaned reads from each library were assembled independently using Trinity tool. Inchworm assembly of reads, the first step of Trinity, resulted in 105,377 and 114,265 contigs with mean sizes of 359 bp and 367 bp, and N50s of 739 bp and 783 bp, for W and R, respectively. After clustering with the TGICL software [33], the contigs were assembled into 69,540 unigenes for W with a mean length of 510 bp and an N50 of 763 bp, and 66,917 unigenes for R with a mean length of 597 bp and an N50 of 1,020 bp. At last, these two sets of unigenes were merged with TGICL resulting in a final assembly of 57,331 All-unigenes (with a total length of ~42 Mb), with a mean size of 739 bp and an N50 of 1,209 bp (Additional file 1). The N50 value is one of the most popular metrics to assess assembly quality, which reflects a continuous and complete assembly [34]. The N50 value of the cranberry fruit transcriptome is longer than that reported in previous studies on blueberry fruit transcriptomes (1,100 bp) [35]. The unigene size distribution showed the following: 76.1 % (43,643) of the unigenes were between 300 and 1,000 bp in length; 22.2 % (12,720) of the unigenes were between 1,000 and 3,000 bp; and 1.7 % (969) were more than 3,000 bp long (Fig. 1). The assembled unigenes were also mapped to the cranberry genome to examine the accuracy of the transcriptomes. 47,316 (82.5 %) unigenes were mapped to the cranberry genome sequence using the NCBI Mega BLAST program [36]. The remaining unigenes that did not map to the cranberry genome may be owing to differences between cultivars, gaps in the genome sequence or too short exons.

**Table 1:** Overview of the sequencing and assembly

	W	R	All
Total raw reads	59,986,374	59,690,570	
Total clean reads	52,413,112	53,352,808	
Total clean nucleotides (bp)	4,717,180,080	4,801,752,720	
Q20 percentage	97.86 %	97.93 %	
GC percentage	46.93 %	47.24 %	
Step-wise assembly			
Contig			
Total number of contigs	105,377	114,265	
Length of all contig (bp)	37,852,028	41,902,139	
Average length of contigs (bp)	359	367	
Contig N50 (bp)	739	783	
Unigene			
Total number of unigenes	69,540	66,917	57,331
Length of all unigene (bp)	35,488,277	39,943,235	42,391,141
Average length of unigenes (bp)	510	597	739
Unigene N50 (bp)	763	1,020	1,209

### Functional annotation by searching similarity

**Fig. 2:**
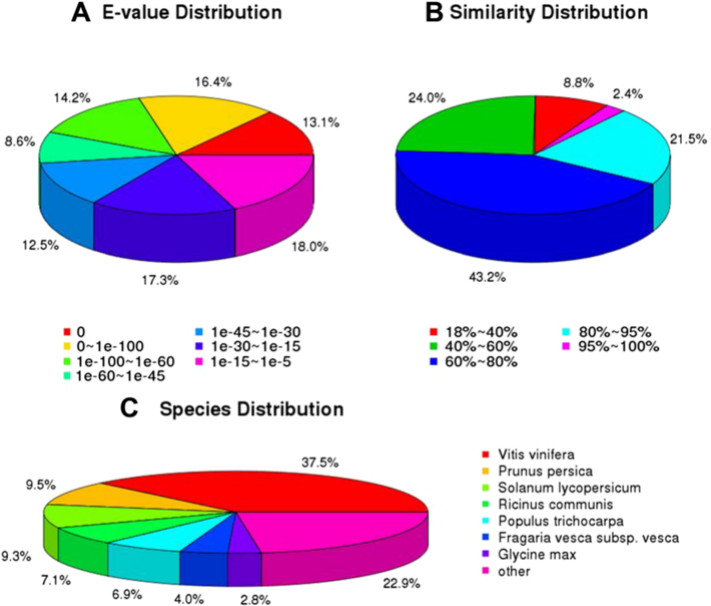
Distribution of the homology search of unigenes against the non redundant (NR) protein database. **a** E-value distribution of the top Blastx hits against the NR database for each unigene; (**b**) Similarly distribution of the top Blastx hits against the NR database for each unigene; (**c**) Species distribution of unigenes matching the top seven species using Blastx in the NR database

For validation and annotation of the assembled unigenes, all unigenes were aligned against the NCBI non-redundant protein (NR), Swiss-Prot protein, Kyoto Encyclopedia of Genes and Genomes (KEGG), Clusters of Orthologous Group (COG) and Gene Ontology (GO) databases using BLASTx, and the nucleotide database (NT) by BlastN with an E-value threshold of 1e-5. A total of 38,460 unigenes (67.08 %) could be matched to known genes in the public databases. This suggests that many genes of unknown function play important roles during cranberry fruit development, and that this process is unexpectedly complex (Table 2). The identity distribution and species distribution were analyzed (Fig. 2). According to the E-value distribution of the top hits in the NR databases, 52.3 % of the matched sequences showed strong homology (<1e-45), while 47.8 % of the matched sequences showed moderate homology (between 1e-5 and 1e-45) (Fig. 2a). For the similarity distribution of the predicted proteins, 67.1 % of the sequences had a similarity higher than 60 %, whereas 32.8 % showed similarity between 18 % and 60 % (Fig. 2b). The species distribution of the top BLAST hits against the NR database for the cranberry fruit transcriptome showed that these unigenes had the greatest number of matches with genes of *Vitis vinifera* (37.5 %), followed by other species including *Prunus persica* (9.5 %), *Solanum lycopersicum* (9.3 %), *Ricinus communis* (7.1 %), *Populus trichocarpa* (6.9 %), *Fragaria vesca subsp. vesca* (4.0 %) and *Glycine max* (2.8 %) (Fig. 2c). The other 22.9 % unigenes had first hits with other species. This indicates that the transcriptome of *V. macrocarpon* is more closely related to that of *V. vinifera* than to other plant genomes that are present in current public databases. In summary, the annotation results suggest that the HiSeq 2000 sequencing project in this study generated a substantial number of assembled transcripts of cranberry fruits.

**Table 2:** Statistics of annotation results against the public databases

Public databases	Number of unigenes	Percentage of unigenes
NR	36,411	63.51 %
NT	29,884	52.13 %
Swiss-Prot	24,033	41.92 %
KEGG	21,898	56.94 %
COG	14,758	38.20 %
GO	27,286	47.59 %
All	38,460	67.08 %

### Functional classification by GO and COG

**Fig. 3:**
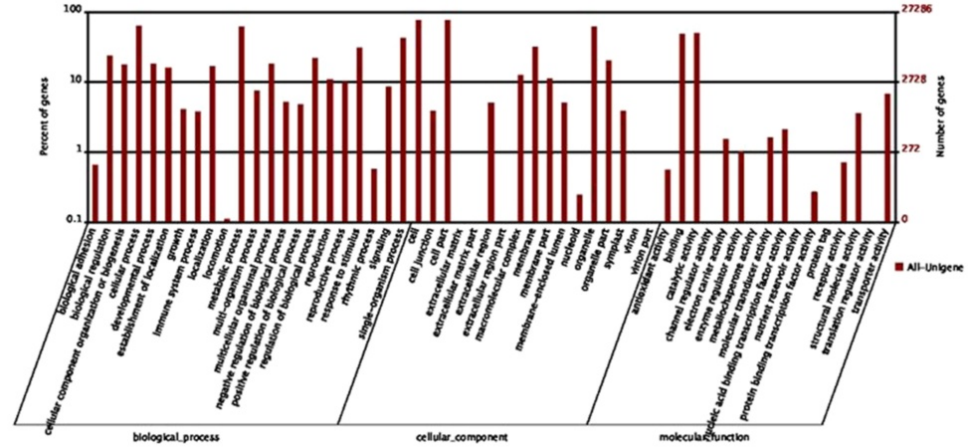
Histogram of GO analysis of All-Unigenes

GO is a useful program for annotating and analyzing the functional categorization of annotated genes. To facilitate the organization of the cranberry transcripts into putative functional groups, GO terms were assigned using Blast2GO [37]. A total of 27,286 unigenes (47.59 %) were assigned into GO ontologies based on their similarity to sequences with previously known functions, including 31,474 sequences assigned to the molecular function category, 104,552 to the biological process category and 83,221 to the cellular component category (Table 2 and Fig. 3). The assigned sequences were divided into 55 functional subcategories. Because 86 % of the unigenes were assigned to more than one GO term, the total number of GO terms was larger than the total number of the unigenes with GO assignments. “Cellular process”, “cell” and “catalytic activity” were the dominant subcategories in the biological process, cellular component and molecular function categories, respectively. Moreover, “metabolic process”, “single-organism process”, “cell part”, “organelle” and “binding” were also well represented, which suggests that many novel genes involved in extensive metabolic activities could be playing important roles during the growth and development stages of the fruit. However, few genes were assigned to the categories “locomotion”, “extracellular matrix”, “extracellular matrix part”, “extracellular region part”, “virion”, “virion part”, “channel regulator activity”, “metallochaperone activity”, “nutrient reservoir activity”, “protein tag”, and “translation regulator activity”. Overall, these GO terms account for almost half of the overall unigenes in the cranberry fruit transcriptomic dataset, suggesting that a large number of cranberry unigenes may be conserved across different plant species.

**Fig. 4:**
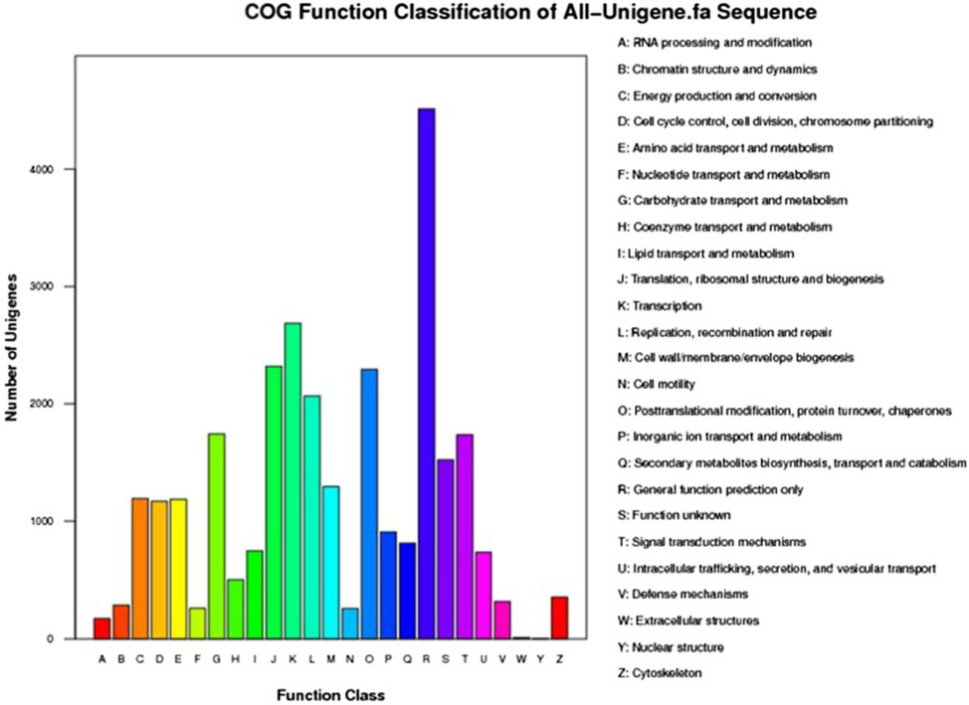
Histogram of COG classification of All-Unigenes

The COG database can phylogenetically predict and classify the completeness of the unigene sequences. Based on sequence homology, 14,758 unigenes (25.7 % of all the unigenes) were assigned into 25 COG categories (Fig. 4). “General function prediction only” represented the largest group (4,511, 15.5 %), followed by “transcription” (2,690, 9.24 %), “translation, ribosomal structure and biogenesis” (2,317, 7.96 %) and “posttranslational modification, protein turnover, chaperones” (2,292, 7.87 %). “RNA processing and modification” (174, 0.60 %), “extracellular structures” (11, 0.04 %) and “nuclear structure” (4, 0.01 %) were the smallest groups. It is noteworthy that 816 unigenes (5.5 %) were classified into the group of “secondary metabolites biosynthesis, transport and catabolism”, suggesting that those secondary metabolite processes play important roles in cranberry fruit development.

### KEGG pathway mapping

KEGG analysis can help us further understand the specific processes, gene functions and gene interactions at a transcriptome level. In our study, 21,898 (38.2 %) all-unigenes were mapped to 128 predicted metabolic pathways through the KEGG database (Table 2 and Additional file 2). The largest category was metabolic pathways (5,522) which included biosynthesis of secondary metabolites (2,603), glycerophospholipid metabolism (973), ether lipid metabolism (824), starch and sucrose metabolism (578), purine metabolism (574), pyrimidine metabolism (501) and other subcategories. In the secondary metabolism category, 31 subcategories comprised 2,603 unigenes, the most represented of which were phenylpropanoid biosynthesis (313), flavonoid biosynthesis (228), limonene and pinene degradation (192), stilbenoid, diarylheptanoid and gingerol biosynthesis (169), terpenoid backbone biosynthesis (150), zeatin biosynthesis (136), carotenoid biosynthesis (130), and flavone and flavonol biosynthesis (108). Moreover, isoflavonoid biosynthesis (53), folate biosynthesis (34), anthocyanin biosynthesis (26), and betalain biosynthesis (6) were also classified. These results suggest that the secondary metabolic processes are active pathways in cranberry fruit development. In addition to metabolism pathways, genes involved in genetic information processing (6,781) and cellular processes (1,718) were highly represented categories. Endocytosis, ribosome, RNA transport, spliceosome, protein processing in endoplasmic reticulum, ribosome biogenesis in eukaryotes, RNA degradation, mRNA surveillance pathway, ubiquitin mediated proteolysis, RNA polymerase, phagosome and peroxisome were included in these categories.

### Candidate genes involved in flavonoid biosynthesis

Given that cranberries are rich in flavonoids, we focus on identifying the candidate genes involved in flavonoid biosynthesis. In plants, flavonoids are a group of polyphenolic secondary metabolites, which play many diverse physiological functions [38]. The flavonoid biosynthetic pathway has largely been characterized in *Arabidopsis thaliana*, *Zea mays*, and *V. vinifera* [39–41]. However, the overall molecular mechanism of flavonoid biosynthesis and accumulation in the cranberry is not fully understood.

**Fig. 5:**
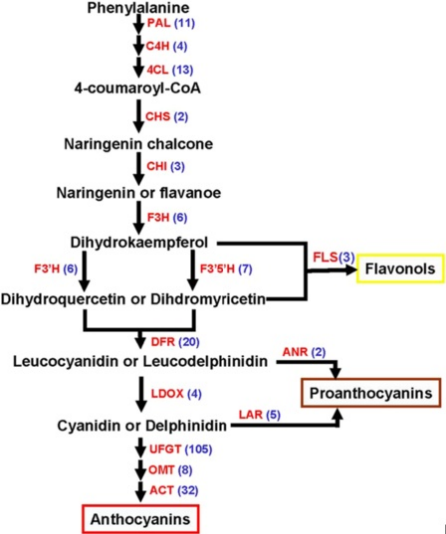
A schematic representation of the anthocyanin biosynthetic pathway emphasizing the anthocyanins, proanthocyanidins and flavonols found in blueberry. Enzyme abbreviations: PAL, phenylalanine ammonia lyase; C4H, cinnamate 4-hydroxylase; 4CL, 4-coumarate CoA ligase; CHS, chalcone synthase; CHI, chalcone isomerase; F3H, flavonoid 3-hydroxylase; F3′H, flavonoid 3′-hydroxylase; F3′5′H, flavonoid 3′,5′-hydroxylase; DFR, dihydroflavonol 4-reductase; LDOX, leucoanthocyanidin dioxygenase; FLS, flavonol synthase; LAR, leucoanthocyanidin reductase; ANR, anthocyanidin reductase; UFGT, UDP-glucose flavonoid 3-*O*-glucosyl transferase; OMT, O-methyltransferase; ACT, anthocyanin acyltransferase

Multiple transcripts encoding almost all known enzymes involved in flavonoid biosynthesis were identified in the annotated cranberry fruit transcriptome. A brief schematic is shown in Fig. 5, which is modified version from Jaakola’s figure [42]. The biosynthesis of anthocyanins, PAs and flavonols shares the upstream phenylpropanoid pathway activated by a cytosolic multienzyme complex [43, 44]. In particular, some of these enzymes belong to the cytochrome-P450 family [45, 46]. First, phenylalanines are converted to chalcones via the phenylpropanoid pathway by the enzymes phenylalanine ammonia lyase (PAL, 11 unigenes), cinnamate 4-hydroxylase (C4H, 4 unigenes), 4-coumarate CoA ligase (4CL, 13 unigenes) and chalcone synthase (CHS, 2 unigenes). Subsequently, chalcone isomerase (CHI, 3 unigenes) catalyzes the stereo-specific cyclization of chalcones into naringenins or flavanoes; flavanone 3-hydroxylase (F3H, 6 unigenes) catalyzes the hydroxylation of flavanones to form the dihydrokaempferols that are continually converted to dihydroquercetins by flavonoid 3′-hydroxylase (F3′H, 6 unigenes), or converted to dihydromyricetins by flavonoid 3′,5′-hydroxylase (F3′5′H, 7 unigenes). Finally, flavonol synthase (FLS, 3 unigenes) catalyzes the conversion of dihydrokaempferols, dihydroquercetins and dihydromyricetins to flavonols.

In the anthocyanin branch, the dihydroquercetins and dihydromyricetins are converted to leucocyanidins or leucodelphinidins by dihydroflavonol 4-reductase (DFR, 20 unigenes). Leucoanthocyanidin dioxygenase (LDOX, 4 unigenes), known as anthocyanidin synthase (ANS), converts leucocyanidins or leucodelphinidins to cyanidins or delphinidins, that are the first colored compounds in the anthocyanin pathway. The final modification steps in the anthocyanin pathway are glycosylation by UDP-glucose flavonoid 3-*O*-glucosyl transferase (UFGT, 105 unigenes), methylation by O-methyltransferase (OMT, 8 unigenes) and acylation by anthocyanin acyltransferase (ACT, 32 unigenes). The synthesis of PAs branches off the anthocyanin pathway after the reduction of leucoanthocyanins (or anthocyanidins) to catechins (or epicatechins). PAs, also known as condensed tannins, are oligomeric or polymeric phenolics that result from the polymerization of flavan-3-ol units. Leucoanthocyanidin reductase (LAR) and anthocyanidin reductase (ANR) are both key enzymes of PA biosynthesis. For the PA pathway, the formation of flavan-3-ols (2,3-*cis*-(−)-flavan-3-ols and 2,3-*trans*-(+)-flavan-3-ols) is achieved by LAR (5 unigenes) and ANR (2 unigenes).

When comparing the cranberry transcriptome to the blueberry fruit transcriptome [35], we found that the cranberry fruit transcriptome data show more abundant flavonoid biosynthesis enzymes (Table 3), especially the number of UFGT enzymes. This suggests that the cranberry contains diverse flavonoid compounds, which have different chemical, size, three-dimensional shape, and physical and biochemical properties. In a previous study that carried out cranberry genome assembly and generated a leaf transcriptome dataset, it was suggested that the cranberry might be lacking two enzymes, F3′5′H and LAR [24]. However, we found seven unigenes encoding F3′5′H and five unigenes encoding LAR in the cranberry fruit transcriptome. Given that flavonoid biosynthesis has temporal and spatial properties, RNA-Seq data of special organs or tissues from different growth and developmental stages could dissect out the complexities involved in flavonoid biosynthesis. Therefore, our cranberry fruit transcriptome undoubtedly provides a powerful supplement to the previous cranberry genome and leaf transcriptome dataset. Furthermore, the metabolomics results showed that the American cranberry contains small amounts of Malvidin, Pelargonidin, Delphinidin, Petunidin, (+)-catechin and (epi)gallocatechins [6, 7]. Therefore, it is not surprising that F3′5′H and LAR are expressed in cranberry fruit.

**Table 3:** The numbers of candidate genes involved in flavonoid transport in the cranberry fruit transcriptome

Family	Number of annotated sequences
ATP-binding cassette (ABC) transporter	341
H^+^-ATPases	174
glutathione S-transferase (GST)	78
multidrug and toxic compound extrusion (MATE)	75
H^+^-PPases	41
soluble *N*-ethylmaleimide-sensitive factor attachment protein receptors (SNARE)	30
vacuolar sorting receptor (VSR)	6

### Candidate genes involved in flavonoid transport

According to previous research performed in plant species including *Arabidopsis* and grape, two mechanisms have been proposed to explain both flavonoid transport from the ER to the vacuole, and the reverse transport from storage sites to other cell targets [47]. Flavonoids could be accumulated into vacuoles or cell walls by the membrane transporter-mediated transport (MTT) system. The proton gradient between the cytosol and the vacuole (or cell wall) by H^+^-ATPases (and H^+^-PPases in the tonoplast) has been proposed to be the main driving force for the transport of some flavonoids [48]. ATP-binding cassette (ABC) transporters have also been claimed to play a role in sequestration of flavonoids into the vacuole [49–51]. In particular, multidrug and toxic compound extrusion protein (MATE) transporters have been described as participating in flavonoid vacuolar sequestration in the tomato, *Arabidopsis* and grapes [48, 52, 53]. Besides the MTT system, the membrane vesicle-mediated transport (MVT) mechanism could also be involved in flavonoid accumulation. The MVT mechanism involves flavonoid-containing vesicles releasing their content into the accumulation targets by fusion [54]. These vesicles require vacuolar sorting receptor (VSR) proteins and soluble *N*-ethylmaleimide-sensitive factor attachment protein receptors (SNARE) proteins to be addressed to the correct compartment and fuse to the membrane target [47]. Additionally, glutathione S-transferase (GST) gene has also been demonstrated to catalyze the conjugation of the tripeptide glutathione (c-Glu-Cys-Gly, GSH) as flavonoid binding proteins, and also be responsible for vesicle uploading or vacuolar transport [55, 56]. In the cranberry fruit transcriptome, 341 unigenes encoding ABC, 75 unigenes encoding MATE, 174 unigenes encoding H^+^-ATPases and 41 unigenes encoding H^+^-PPases were identified. Moreover, 78 unigenes encoding GST, 6 unigenes encoding VSR and 30 unigenes encoding SNARE were found. These results imply that the two distinct transport mechanisms (MTT and MVT) could both be present, and transport may be a multifactorial process in the cranberry, involving different strategies and the contribution of several enzymes. Unlike similar studies in the carnation and the grape vine, we did not find bilitranslocase (BTL), which is a putative flavonoid that is localized in the liver and gastric mucosa in mammals [57, 58]. These findings provide a powerful genomic tool to research the mechanisms of transport and accumulation of flavonoids in the cranberry.

### Candidate transcription factors involved in flavonoid biosynthesis and transport

The main structural genes encoding enzymes involved in the flavonoid biosynthetic pathway have been studied in many species, including *Arabidopsis*, maize, the petunia, the snapdragon, apples and grapes [59]. In particular, the expression of structural genes involved in flavonoid synthesis is largely controlled by basic helix–loop–helix (bHLH) transcription factors (TFs), MYB proteins and WD-repeat-containing proteins [60, 61]. bHLH TFs belong to multigenic families and are structurally organized into basic helix-loop-helix DNA-binding conserved motifs [62, 63]. MYB TFs are a large gene family that is thought to be one of the most important plant regulatory gene families [64, 65]. In plants, MYB TFs have been demonstrated to be involved in control of phenylpropanoid secondary metabolism, including the biosynthesis of flavonoids and anthocyanins [66–69]. WD-repeat-containing proteins consist of four or more copies of the WD (tryptophan-aspartate) repeat, which is a sequence motif approximately 31 amino acids long that encodes a structural repeat [70]. It has been reported that certain MYB TFs interact with bHLH and WD40 proteins to form a MYB-bHLH-WD40 complex in the regulation of the flavonoid biosynthetic pathway [71, 72]. Moreover, TFs also control the regulation of flavonoid transport. For example, *Arabidopsis* R2R3-MYB TF (AtTT2) regulates the expression of the MATE transporter gene *TT12* to control the flavonoids in developing siliques; and the maize ABCC transporter protein *ZmMRP3* is regulated by the R (bHLH family) and C1 (R2R3-MYB) TFs to control anthocyanin transport [73, 74]. Based on our results, a total of 669 unigenes were identified as putative TFs or regulators, with 641 of the unigenes belonging to 65 subclasses (Table 4). The unigenes encoding WD40 (202), bHLH (53), MYB (41) and WRKY (41) are the most abundant in the cranberry fruit transcriptome. By comparison to the number of WD40 unigenes detected in the blueberry fruit transcriptome, the cranberry shows more than twice the number of WD40 unigenes. These results imply that the WD40 proteins may be playing an important role in the regulation of flavonoid biosynthesis and transport. Because all major TF subclasses known to be involved in regulating anthocyanin biosynthesis are highly expressed in the cranberry fruit transcriptome, we conclude that flavonoid biosynthesis and transport are regulated by TFs in the cranberry, such as WD40, bHLH, MYB and WRKY. The control mechanism of flavonoid biosynthesis and transport is complex in the cranberry, and may be independently regulated by a single TF, or controlled by combinations of TF complexes.

**Table 4:** The numbers of transcription factors involved in flavonoid biosynthetic and transport in the cranberry fruit transcriptome

Family	Number of annotated sequences	Family	Number of annotated sequences
WD40	202	RNA polymerase II	3
bHLH	53	UNE	3
MYB	41	CRF	2
WRKY	41	global	2
GATA	28	HD	2
TCP	19	ICE	2
bZIP	18	jumonji domain-containing	2
ERF	18	MYC	2
nuclear TF	18	RAP	2
GTE	17	RAV	2
GRAS	16	RAX	2
AP2	15	sfc	2
HSF	10	WER	2
trihelix	10	WRI	2
BTF	8	AS	1
ccaat-binding	7	COL	1
PIF	7	CPP	1
TGA	7	DOF	1
ILR	6	DPB	1
NAC	5	DRE	1
RF	5	yellow 2	1
ASG	4	HY5	1
BIM	4	IIIB	1
general	4	KNOTTED	1
HBP	4	m50	1
KAN	4	NAP	1
scarecrow	4	Pur-alpha	1
APETALA	3	RWP-RK	1
B3	3	SPATULA	1
CPC	3	UPBEAT	1
E2F	3	VIP	1
MADS-box	3	YABBY	1
ORG	3	others	28

### SSR motif discovery

SSRs have been widely used in the study of genetic identification and fingerprint mapping, due to their high polymorphic information content, simple technology, and high reproducibility. The transcriptome is also an important resource for rapid and cost-effective development of genetic markers. To further evaluate the quality of assembly and develop new molecular markers, the cranberry unigenes generated in this study were used to identify SSRs. The distribution of mono-, di-, tri-, quad-, penta- and hexa-nucleotide SSRs in these unigenes are shown (Table 5). A total of 14,473 SSRs were identified in 11,980 unigenes. Of the11,980 unigenes, 2,215 and 851 unigenes contained more than one SSR and SSRs in compound formation, respectively. This indicates that nearly 21 % of the cranberry unigenes contained SSRs, which is higher than the SSR ratio (8.6 %) reported in the cranberry leaf transcriptome, but lower than the SSR ratio (37.8 %) in the cranberry genome [24]. The largest fraction of SSRs identified was di-nucleotide repeats (62.4 %), followed by tri- (17.4 %) and mono- repeats (17.3 %). The di-nucleotide repeats were the most abundant type, accounting for 62.4 %, which is higher than the findings in the cranberry genome (44 %) and the leaf transcriptome (35 %) [24]. SSR motifs were further analyzed for the number of repeating units, and the most represented repeats of potential SSRs was nine (Additional file 3). The most frequent mono-, di-, tri-, quad-, and penta-nucleotide motifs were A, GA, GAA, AAAG and AAAAG, accounting for 8.7 %, 18 %, 1.4 %, 0.3 % and 0.8 %, respectively. These results show that the SSRs identified in the cranberry fruit transcriptome were GA rich, which is similar to the earlier reports on the cranberry genome (16.5 %) and the leaf transcriptome (15 %). The SSRs identified in the present study provide an abundant resource for molecular marker studies in the cranberry.

**Table 5:** Summary of SSR identified in cranberry fruit transcriptome

Searching item	Numbers
Total number of sequences examined	57,331
Total size of examined sequences (bp)	42,391,141
Total number of identified SSRs	14,473
Number of SSR containing sequences	11,980
Number of sequences containing more than 1 SSR	2,115
Number of SSRs present in compound formation	851

### SSR marker validation

**Fig. 6:**
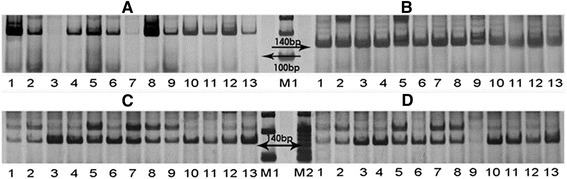
Silver stained polyacrylamide gels of four SSRs on CL2296.Contig1 **a**, CL284.Contig2 (**b**), CL2216.Contig1 (**c**) and CL2216.Contig4 (**d**); PCR products from line1 to line13 are Pilgrim, Stankawich, Howes, Bergmen, WAU108, Brewer, Bain Fav. No.1, Bain11, Le Muryon, Hollister Red, Mathewes, Bain6 and Washington, respectively. (M1: Marker DL2000; M2: Marker DL100)

We designed primer pairs for 12 unigenes from the GO term “response to stimulus/chemicals”. The sequences of the primers are in Additional file 4. Silver stained polyacrylamide gels results showed that 11 pairs (91.7 %) successfully amplified bands. Among the 11 primer pairs, four primer pairs that showed polymorphic bands and seven that showed monomorphic bands. The four polymorphic SSR loci are shown in Fig. 6. The primers for CL2296.Contig2 amplified bigger band (~200 bp) than expected size (Fig. 6a), that may be due to the regions between the two primers contain introns. The primers for CL284.Contig2 amplified expected band (Fig. 6b), the primers for CL2216.Contig1 and CL2216.Contig4 both amplified two alleles (Fig. 6c, d). Thus, these results indicate that the novel SSR primers show good transferability to cultivars of cranberry. However, the polymorphic rate is low owing to the conserved nature of expressed sequence tag SSRs (EST-SSRs) [75]. Therefore, the novel primers developed may represent highly-conserved genes involved in the response to stimuli/chemicals, which would be useful for the breeding of resistant cranberry cultivars and other *Vaccinium* plants in future studies.

### Difference in gene expression between white and red fruit

**Fig. 7:**
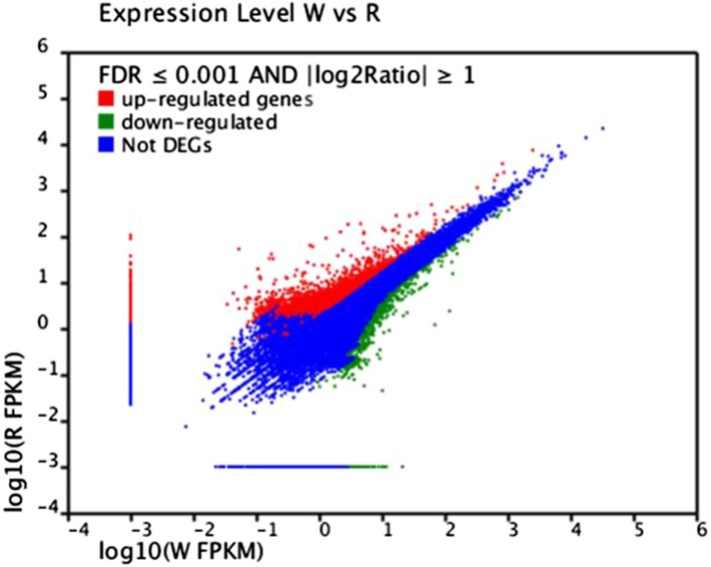
Gene transcription profile between W and R libraries. For comparing gene expression level between the two libraries, each library was normalized to 1 million tags. Red dots represent transcripts more prevalent in R library, green dots show those present at a lower frequency in W library and blue dots indicate transcripts that did not change significantly

**Fig. 8:**
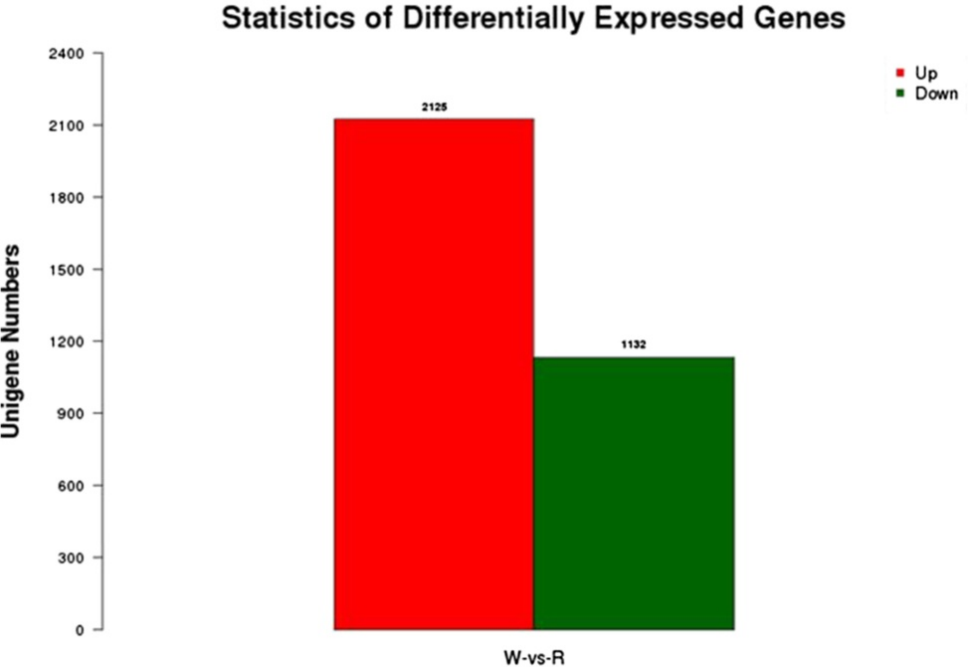
The number of up-regulated and down-regulated genes between W and R libraries

To obtain a digital expression profile of the differentially expressed genes between the two fruit development stages in the cranberry, we used the FPKM (Fragments per kb per million fragments) method to perform gene expression analysis between the W and R libraries [76]. The transcripts with at least a two-fold difference between white and red fruits are shown in Fig. 7. A total of 3,257 unigenes were identified as differentially expressed genes (DEGs) between the two developmental stages of cranberry fruit (Fig. 8). Among them, 2,125 and 1,132 unigenes were highly expressed in the red and white fruits, respectively. More genes were highly expressed in red fruits, suggesting that more genes were involved in complex metabolites during the full ripening stage of the fruit. Moreover, 3,010 and 1,328 unigenes were only expressed in red and white fruits, respectively. This indicates that the unigenes may be specifically expressed in the different development stages of cranberry fruits.

**Fig. 9:**
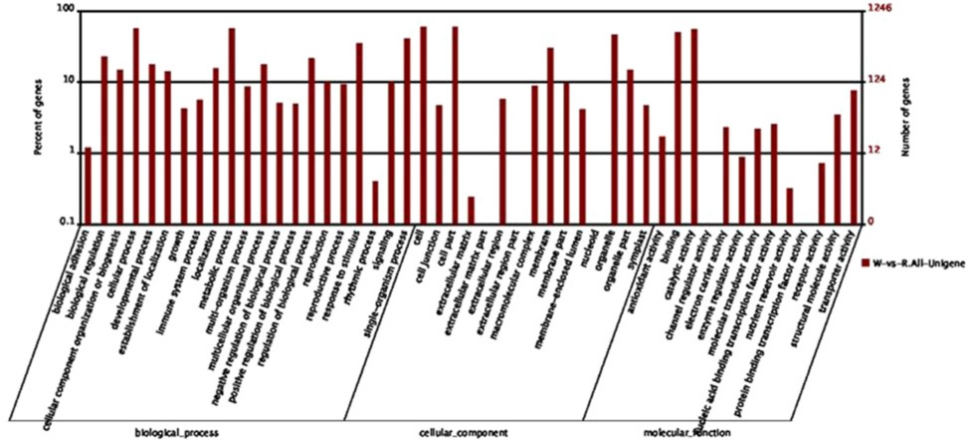
Histogram of GO analysis of DEGs

We identified 1,246 DEGs that were assigned to 49 significantly enriched GO terms. The most abundant GO terms were “metabolic process”, “cell and cell part” and “catalytic activity” in the biological process, cellular component and molecular function categories, respectively (Fig. 9). Moreover, there are some interesting biological processes which are associated with fruit development, such as “developmental process”, “growth”, “reproduction”, “reproductive process”, “antioxidant activity” and “transporter activity”.

We also found that a total of 113 pathways were affected by 1,134 DEGs (Additional file 5). Notably, the greatest number of DEGs were mapped to “metabolic process” (384, 33.86 %) and “biosynthesis of secondary metabolites” (202, 17.81 %). In secondary metabolites biosynthesis, 39 (3.44 %) DEGs are mapped to “phenylpropanoid biosynthesis”, 28 (2.47 %) DEGs to flavonoid biosynthesis, 15 (1.32 %) DEGs to flavone and flavonol biosynthesis, 8 (0.71 %) DEGs to benzoxazinoid biosynthesis, 16 (1.41 %) DEGs to stilbenoid, diarylheptanoid and gingerol biosynthesis, 1 (0.09 %) DEG to betalain biosynthesis, 3 (0.26 %) DEGs to isoquinoline alkaloid biosynthesis, 3 (0.26 %) DEGs to tropane, piperidine and pyridine alkaloid biosynthesis, 1 (0.09 %) DEG to anthocyanin biosynthesis, and 2 (0.18 %) DEGs to isoflavonoid biosynthesis. This would reflect a higher specialization in partitioning the secondary metabolism intermediates towards different classes of end derivatives which accumulate in the same organ during the different stages of berry development.

**Fig. 10:**
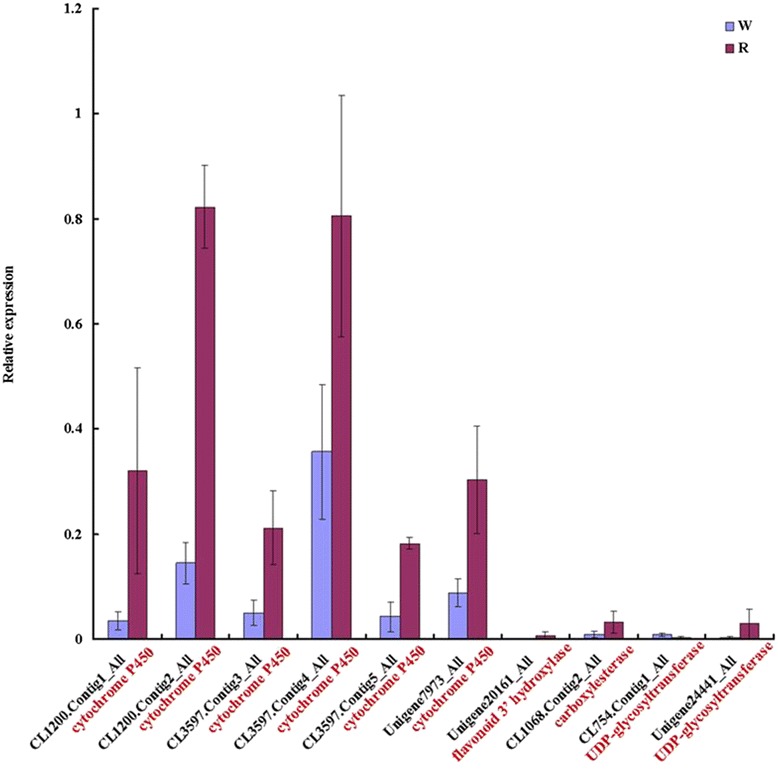
The expression profiles of 10 unigenes between W and R libraries in cranberry

To validate the data from our digital expression analysis, quantitative Real Time PCR (qRT-PCR) assays were performed on 10 DEGs involved in the phenylpropanoid biosynthesis, flavone and flavonol biosynthesis, and flavonoid biosynthetic pathways (Table 6). The qRT-PCR expression pattern of these unigenes is shown in Fig. 10. Except for the unigenes encoding UDP-glycosyltransferase (CL754.Contig1_All), cytochrome P450 (CL1200.Contig1_All, CL1200.Contig2_All, CL3597.Contig3_All, CL3597.Contig4_All, CL3597.Contig5_All and Unigene7973_All), flavonoid 3′ hydroxylase (Unigene20161_All), carboxylesterase (CL1068.Contig2_All), and UDP-glycosyltransferase (Unigene 24441_All), the unigenes were up-regulated in the red fruit stage. The results of the qRT-PCR are consistent with our digital expression data (Table 6). This also showed an association with flavonoid production, and it is likely that these genes are involved in the fruit development stage. Plant cytochrome P450s are a large superfamily of heme-containing monooxygenases, which are involved in a wide range of secondary metabolite biosynthetic reactions, such as terpenoids, phenylpropanoids, and nitrogen-containing compounds, including alkaloids, cyanogenic glucosides, and glucosinolates [77]. Therefore, the selected unigenes encoding cytochrome P450s may play important roles in flavonoid accumulation in cranberry fruit.

**Table 6:** All-unigene selected for qRT-PCR in Illumina/Solexa sequencing library of cranberry fruit

Gene ID	Gene Length	W_FPKM	R_FPKM	log2 Ratio	Up/Down	Nr-annotation
CL1200.Contig1_All	2042	6.2174	34.4668	2.4708	Up	cytochrome P450
CL1200.Contig2_All	930	1.421	23.6309	4.0557	Up	cytochrome P450
CL3597.Contig3_All	343	7.7056	56.6377	2.8778	Up	cytochrome P450
CL3597.Contig4_All	2327	1.1155	2.909	1.3828	Up	cytochrome P450
CL3597.Contig5_All	2143	1.894	5.2357	1.4669	Up	cytochrome P450
Unigene7973_All	1103	15.7038	55.2759	1.8155	Up	cytochrome P450
Unigene20161_All	1637	4.0652	12.8019	1.655	Up	flavonoid 3'-hydroxylase
CL1068.Contig2_All	200	3.0678	9.9684	1.7002	Up	carboxylesterase 2-like
CL754.Contig1_All	628	5.0353	11.5911	1.2029	Up	UDP-glycosyltransferase
Unigene24441_All	654	0.7938	6.2386	2.9744	Up	UDP-glycosyltransferase

## Conclusions

A high-quality transcriptomic dataset of the cranberry fruit was obtained using the Illumina HiSeq 2000 sequencing platform. A significant number of important metabolic pathways and functions associated with the unigenes were identified. Moreover, a large number of SSRs were identified that can be used for subsequent marker development, genetic linkage and QTL analysis. Many candidate genes that are potentially involved in flavonoid biosynthesis, transport and regulation were identified and are worthy of further functional research. Our study provides the largest number of unigenes to date, and lays the groundwork for in-depth transcriptomic profiling of cranberry fruits.

## Materials and methods

### Plant material and tissue collection

Six-year-old cranberry plants (*V. macrocarpon* ‘Bergman’) were grown in Changchun, Jilin Province in northeast China. This cultivar is a cross between ‘Early Black’ and ‘Searles’ with a basic chromosome number x = 12 (2x = 2n = 24) [78]. It propagates by asexual propagation of cutting and micro-propagation, which permits homogeny among individual plants. Ten plants were selected as a sample. We removed the weak buds and opened the flowers, keeping the flowers at the full pink bud stage with forceps. The flowers can be thinned to one per stem to ensure a better fruit set. Fruit samples (almost 20 fruits of the same maturation degree) were collected based on the skin color of the fruit and the development period in days. Fruit sampling included two ripening stages, the white fruit (30 days after full bloom, stage W) and red fruit (60 days after full bloom, stage R). For both of the stages, all of the fruit, including pulp, skin and seeds, were mixed, and immediately frozen in liquid nitrogen and kept at −80 °C until they were used for sample preparation.

### Library preparation and sequencing

Total RNA was isolated by TRIzol® Reagent (Invitrogen, Carlsbad, CA, USA). The RNA quantity and quality were assessed by a Nanodrop 2000 instrument (Thermo Scientific, USA) and an Agilent 2100 Bioanalyzer (Agilent Technologies, California, USA). The cDNA libraries were constructed by mixing equal quantities of RNA from each fruit stage, including pericarp, pulp and seeds, according to the manufacturer’s instructions (Illumina, San Diego, CA, USA). First, magnetic beads with oligo(dT) molecules were used to enrich for poly(A) mRNA from 20 μg of total RNA. Then, samples were fragmented into short pieces and reverse transcribed into cDNA with a PrimeScript™ 1st Strand cDNA Synthesis Kit (Takara). The second-strand cDNA was then synthesized using buffer, dNTPs, RNase H, and DNA polymerase I. Short fragments were purified using the QIAquick PCR Purification Kit (QIAGEN) and resolved with EB buffer for end repair and single nucleotide A (adenine) tailing. After this, short fragments were connected with sequencing adapters, and enriched by 15 cycles of PCR amplification to select suitable fragments for the final cDNA library. The quantification and qualification of the cDNA library were characterized by an Agilent 2100 Bioanalyzer and ABI StepOnePlus Real-Time PCR System, and sequenced on the Illumina HiSeq™ 2000 pair-end system. Illumina sequencing was performed at the Beijing Genomics (BGI) Center in Shenzhen, China.

### Sequence assembling and analysis

The raw reads generated from the Illumina sequencing were pre-processed with the filter-fq software (unpublished software). The whole sequences of reads only with adaptor, reads containing more than 5 % unknown nucleotides, and low quality reads (reads containing more than 20 % bases with Q-value ≤ 10) were eliminated. The clean reads were assembled into contigs using the Trinity tool with the following parameters: seqType = fq, min_contig_length = 100, min_glue = 3, group_pairs_distance = 250, path_reinforcement_distance = 85, bfly_opts = ′-V 5 --edge-thr = 0.05 --stderr', min_kmer_cov = 3, CPU = 7 [79]. First, the RNA-Seq data were assembled into longer, gapless unique sequences with the Inchworm software. The resulting sequence from Inchworm was defined as contigs. Subsequently, the contigs were clustered and complete de Bruijn graphs were constructed for each cluster using the Chrysalis software. Then, the Butterfly software was used to track the paths that reads and pairs of reads took within the graphs. As multiple samples from the same species were sequenced, unigenes from each sample’s assembly were taken for further processing with the sequence clustering software TGICL, for sequence splicing and overlap removal to acquire non-redundant unigenes that were as long as possible [33]. At last, the unigenes from each sample were assembled for unique All-unigene sequences by TGICL software. For gene family clustering, the unigenes were divided into two classes: clusters and singletons. The former were prefixed ‘CL’ and the cluster id followed this prefix. In one cluster, the similarity between unigenes was more than 70 %. For the singletons, the prefix was ‘unigene’. To assess the quality of the *de novo* assembly, a similarity search against the cranberry genome was further conducted using the Mega BLAST algorithm with a score greater than 200 [36].

All unigene sequences were aligned by BLASTx (E-value < 0.00001) to protein databases such as NR, Swiss-Prot, GO, COG and KEGG, and to NT using the BlastN (E-value < 0.00001), retrieving proteins with the highest sequence similarity with the given unigenes, along with their protein functional annotations [80, 81]. When a unigene was not aligned to any of the above databases, ESTScan software was used to decide upon the sequence direction [82]. For NR annotation, the Blast2GO program was used to get the GO annotation of unigenes [37]. Then, WEGO software was used to conduct GO functional classification for all unigenes and to understand the distribution of the gene functions [83].

### SSR detection and validation

MicroSAtellite (MISA) (http://pgrc.ipk-gatersleben.de/misa/misa.html) was used to identify SSRs in the assembled transcriptome of the cranberry fruit. SSR motifs were identified between one and six nucleotides in size and with 1, 2, 3, 4, 5 and 6 repeats for mono-, di-, tri-, quad-, penta-, and hexamers, respectively. The sequences with a minimum of 150 bp flanking region from SSR loci were used to design primers using PRIMER 3.0 software [84]. The major primer design parameters were as follows: a PCR product length of 80–300 bp; a primer size of 18–28 bp (optimum 23 bp); a melting temperature of 51–58 °C (optimum 60 °C); and a temperature difference between the forward primer and reverse primer less than 2 °C. Then, the final primers were obtained by filtering rules as follows: no SSRs in the primer; align the primers to the unigene sequence with the 5′ site allowed three mismatches and the 3′ site allowed one mismatch; remove the primers which aligned to more than one unigene; find SSRs on the product sequences by ssr_finder (http://www.fresnostate.edu/ssrfinder/) and keep the product for which the result of ssr_finder is the same as the result of MISA.

Thirteen cranberry cultivars were used to validate the SSR markers by PCR. Fresh leaves were collected for genomic DNA extraction by the cetyltrimethylammonium bromide (CTAB) method [85] with a minor modification. The PCR reaction was performed in a 20 μl reaction volume containing 2 μl of 10× PCR Buffer, 0.4 μl of dNTPs (10 mmol/L), 0.4 μl of genomic DNA (100 ng/μl), 0.8 μl of both the forward primer and reverse primer (10 μM), and 0.1 μl of Taq polymerase (5 U/μl). The PCR conditions used were as follows: initial denaturation for 2 min at 94 °C, followed by 35 cycles of denaturation for 30 s at 95 °C, annealing for 30 s at 55 °C (primer specific), and extension for 30 s at 72 °C, followed by a final extension for 5 min at 72 °C, ending at 4 °C. The amplification products were separated by 6 % native polyacrylamide gel electrophoresis and were visualized by silver staining.

### Differential gene expression analysis

An analysis of statistical comparison was performed to predict genes with different expression levels using the method previously described by Audic and Claverie [86]. The calculation of unigene expression used the FPKM method and SOAP (http://soap.genomics.org.cn/soapaligner.html), which were able to eliminate the influence of different gene lengths and sequencing levels on the calculation of gene expression [76]. Therefore, the calculated gene expression could be directly used to compare the difference in gene expression between the samples. The FPKM formula was:

$$ FPKM=\frac{10^6C}{NL/{10}^3,} $$

where C is the number of cleaned reads that were uniquely aligned to one unigene; N is the total number of cleaned reads that were uniquely aligned to all unigenes; and L is the base number in the CDS (Coding sequence) of one unigene. The expression between two samples can also be assessed with the FDR (False Discovery Rate) method, which is a statistical method used in multiple hypothesis testing to correct for *P*-value [87]. In our analysis, an estimated absolute value of log_2_-fold change of ≥2 and FDR adjusted *P*-value ≤ 0.001 were used as the threshold to judge the significance of DEGs. DEGs were subsequently carried into GO functional analysis and KEGG pathway analysis.

### Confirmation of gene expression by qRT-PCR

Total RNA was extracted as described for the cDNA library preparation and sequencing. Each RNA sample was treated with PrimeScript™ RT Reagent Kit with gDNA Eraser (Takara), to remove residual genomic DNA and reverse transcribe into cDNA, according to the manufacturer’s protocol. The primers for qRT-PCR were designed using the Primer Express® Software v3.0.1 (Applied Biosystems), and are listed in Additional file 6. The actin gene of cranberry (CL7164.Contig2_All) was used as the internal housekeeping gene control. qRT-PCR was performed using the SYBR Premix Ex Taq Kit (Takara) according to the manufacturer’s instructions. The PCR was carried out on a Stratagene Mx3000P instrument (Agilent, USA) with the following conditions: denaturation at 95 °C for 15 s; 40 cycles of 95 °C for 15 s, and 60 °C for 30 s. All qRT-PCR reactions were repeated three times, with three technical replications per experiments. The results were normalized to the expression level of the constitutive actin gene. A relative quantitative method (2^-ΔΔCt^) was used to evaluate the quantitative variation.

## Availability of supporting data

All clean reads generated by Illumina sequencing have been deposited in the Sequence Read Archive (SRA) data base (http://www.ncbi.nlm.nih.gov/sra) under the accession ID SRX1140359 for R, and SRX1140360 for W.

## Additional files

Additional file 1:
**57,331 All-unigene sequences in cranberry fruit transcriptome, available at** **https://mynotebook.labarchives.com/**, **DOI:** 10.6070/H4FB50ZC (FA 44805 kb) Additional file 2:
**21,898 cranberry unigenes assigning to 128 KEGG pathways.** (XLS 50 kb) Additional file 3:
**Frequency of identified SSR motifs.** (XLS 57 kb) Additional file 4:
**Primer sequences of cranberry selected for SSR marker validation.** (XLS 19 kb) Additional file 5:
**1,134 DEGs assigning to 113 KEGG pathways.** (XLS 53 kb) Additional file 6:
**Primer sequences of cranberry selected for qRT-PCR analysis.** (XLS 16 kb)

## Abbreviations

4CL: 4-coumarate CoA ligase
ABC: ATP-binding cassette
ACT: Anthocyanin acyltransferase
ANR: Anthocyanidin reductase
ANS: Anthocyanidin synthase
bHLH: basic helix–loop–helix
BTL: Bilitranslocase
C4H: Cinnamate 4-hydroxylase
CDS: Coding sequence
CHI: Chalcone isomerase
CHS: Chalcone synthase
COG: Clusters of orthologous group
CTAB: Cetyltrimethylammonium bromide
DEGs: Differentially expressed genes
DFR: Dihydroflavonol 4-reductase
F3′5′H: Flavonoid 3′,5′-hydroxylase
F3′H: Flavonoid 3′-hydroxylase
F3H: Flavanone 3-hydroxylase
FDR: False discovery rate
FLS: Flavonol synthase
FPKM: Fragments per kb per million
GO: Gene ontology
GSH: c-Glu-cys-gly
GST: Glutathione S-transferase
HPLC: High-performance liquid chromatography
KEGG: Kyoto encyclopedia of genes and genomes
LAR: Leucoanthocyanidin reductase
LDOX: Leucoanthocyanidin dioxygenase
MATE: Multidrug and toxic compound extrusion protein
MISA: MicroSAtellite
MS: Mass spectrometer
MTT: Membrane transporter-mediated transport
MVT: Membrane vesicle-mediated transport
NGS: Next-generation sequencing
NR: Non-redundant protein
NT: Nucleotide database
OMT: O-methyltransferase
PAL: Phenylalanine ammonia lyase
PAs: Proanthocyanidins
qRT-PCR: Quantitative real time PCR
QTL: Quantitative trait loci
RNA-Seq: RNA sequencing
SNARE: Soluble N-ethylmaleimide-sensitive factor attachment protein receptors
SSRs: Simple sequence repeats
TFs: Transcription factors
UFGT: UDP-Glucose flavonoid 3-O-glucosyl transferase
UV/Vis: Ultraviolet–visible
VSR: Vacuolar sorting receptor
WD: Tryptophan-aspartate
